# Efficacy of Upadacitinib in refractory Polyarteritis Nodosa: a case report

**DOI:** 10.1093/omcr/omae199

**Published:** 2025-02-22

**Authors:** Akihiko Nakabayashi, Erika Iguchi, Dong Seop Kim, Yanakawee Siripongvutikorn, Akira Nishigaichi, Maiko Yoshimura, Hyota Takamatsu, Shiro Ohshima

**Affiliations:** Department of Rheumatology, NHO Osaka Minami Medical Center, 2-1 Kidohigashi, Kawachinagano, Osaka 586-8521, Japan; Department of Rheumatology, NHO Osaka Minami Medical Center, 2-1 Kidohigashi, Kawachinagano, Osaka 586-8521, Japan; Department of Rheumatology, NHO Osaka Minami Medical Center, 2-1 Kidohigashi, Kawachinagano, Osaka 586-8521, Japan; Department of Rheumatology, NHO Osaka Minami Medical Center, 2-1 Kidohigashi, Kawachinagano, Osaka 586-8521, Japan; Department of Rheumatology, NHO Osaka Minami Medical Center, 2-1 Kidohigashi, Kawachinagano, Osaka 586-8521, Japan; Department of Rheumatology, NHO Osaka Minami Medical Center, 2-1 Kidohigashi, Kawachinagano, Osaka 586-8521, Japan; Department of Rheumatology, NHO Osaka Minami Medical Center, 2-1 Kidohigashi, Kawachinagano, Osaka 586-8521, Japan; Department of Clinical Research/Rheumatology, NHO Osaka Minami Medical Center, 2-1 Kidohigashi, Kawachinagano, Osaka 586-8521, Japan; Department of Rheumatology, NHO Osaka Minami Medical Center, 2-1 Kidohigashi, Kawachinagano, Osaka 586-8521, Japan; Department of Clinical Research, NHO Osaka Minami Medical Center, 2-1 Kidohigashi, Kawachinagano, Osaka 586-8521, Japan

**Keywords:** biological therapy, cytokines, Janus kinase inhibitors, polyarteritis nodosa, systemic vasculitis, treatment failure

## Abstract

Polyarteritis nodosa (PAN) is systemic vasculitis, typically treated with a combination of glucocorticoids and immunosuppressants. Changing the immunosuppressant is recommended if remission cannot be achieved with these treatments. However, there is a lack of further treatment options for patients who are unresponsive to all immunosuppressants. We report a 44-year-old Japanese man with refractory PAN who was unresponsive to various immunosuppressants (including tocilizumab). Upadacitinib treatment led to relatively rapid symptom improvement, allowing for tapering and eventual discontinuation of immunosuppressants, including prednisolone. During his 1-year follow-up, no relapse or side effects were noted. This case suggests that Janus kinase inhibitors may provide a breakthrough for patients with refractory PAN.

## Introduction

Polyarteritis nodosa (PAN) is a systemic necrotizing vasculitis that affects medium-sized arteries and is not associated with glomerulonephritis or small vessel involvement. Antineutrophil cytoplasmic antibodies are typically negative in these patients. PAN can be triggered by viral infections, particularly hepatitis B virus (HBV), although the cause is often unknown [1].

For the treatment of nonsevere PAN, a combination of glucocorticoids (GC) and immunosuppressants other than cyclophosphamide (CY) is recommended over GC alone. For severe cases, a combination of CY and GC is preferred over GC alone. When remission cannot be achieved with these treatments, changing the immunosuppressant is often recommended. However, there is a lack of further treatment options for patients who are unresponsive to all immunosuppressants [2].

We report a patient with refractory PAN who did not respond to treatment with azathioprine (AZP), mycophenolate mofetil (MMF), methotrexate (MTX), tacrolimus (TAC), CY, and tocilizumab (TCZ). The use of the Janus kinase (JAK) inhibitor upadacitinib (UPA) led to a relatively quick improvement in symptoms, allowing for the tapering and discontinuation of prednisolone (PSL), MTX, and nonsteroidal anti-inflammatory drugs (NSAIDs). After one year of UPA use, no relapses or side effects have been observed. This case suggests that JAK inhibitors may provide a breakthrough for patients with refractory PAN.

## Case report

The patient was a 44-year-old Japanese man with a history of acute appendicitis at the age of 15 years. He had no known allergies. He was a chronic smoker (15 cigarettes/day for 25 years) and consumed alcohol occasionally. From the age of 20 years, he experienced recurrent painful subcutaneous nodules on his limbs. At the age of 39 years, he developed polyarthritis and myalgia. Blood investigations performed at a local public hospital revealed elevated C-reactive protein (CRP) level. Myeloperoxidase-anti-neutrophil cytoplasmic antibody and proteinase-3-anti-neutrophil cytoplasmic antibody were negative, eosinophil count was normal, and urine tests were unremarkable (Table 1). He had no symptoms affecting the eyes, nose, or mouth. Furthermore, chest computed tomography showed no signs of interstitial pneumonia. He tested negative for HBV.

**Table 1:** Laboratory data before public hospital treatment.

**Complete blood cell** White blood cell count (/μl)	12 510	LDH (U/l) CK (U/L)	359 52
Red blood cell count (/μl)	5.41 × 10^6^	T-Bil (mg/dl)	0.47
Hemoglobin (g/dl)	15.0	TP (g/dl)	8.0
Platelet (/μl)	30.0 × 10^4^	Alb (g/dl)	3.9
**Coagulation test**		ESR (mm/h)	31
APTT (second)	30.6	**Immunochemistry**	
PT-INR	0.95	CRP (mg/dl)	5.50
**Biochemistry**		IgG (mg/dl)	1848
HbA1c (%)	5.7	ANA	<40
BUN (mg/dl)	17.7	Anti-SS-A Ab (U/ml)	<0.5
Cr (mg/dl)	0.81	RF (U/ml)	14.2
eGFR (ml/min/1.73 m^2^)	84.75	ACPA (U/l)	<0.5
Na (mEq/l)	142	MMP-3 (ng/ml)	301.0
K (mEq/l)	4.4	C3 (mg/dl)	172.1
Cl (mEq/l)	106	C4 (mg/dl)	49.7
AST (U/l)	14	CH50 (U/ml)	89.6
ALT (U/l)	17	MPO-ANCA (IU)	<0.5
ALP (U/l)	268	PR3-ANCA (IU)	<0.5

APTT: activated partial thromboplastin time; PT-INR: prothrombin time-international normalized ratio; HbA1c: hemoglobin A1c; BUN: blood urea nitrogen; Cr: creatinine; eGFR: estimated glomerular filtration rate; Na: sodium; K: potassium; Cl: chloride; AST: aspartate aminotransferase; ALT: alanine aminotransferase; ALP: alkaline phosphatase; LDH: lactate dehydrogenase; CK: creatine kinase; T-Bil: total bilirubin; TP: total protein; Alb: albumin; ESR: erythrocyte sedimentation rate; CRP: C-reactive protein; IgG: immunoglobulin G; ANA: antinuclear antibody; Anti-SS-A Ab: anti-SS-A antibody; RF: rheumatoid factor; ACPA: anti-cyclic citrullinated peptide antibody; MMP-3: matrix metalloproteinase-3; C3: complement component 3; C4: complement component 4; CH50: 50% hemolytic unit of complement; MPO-ANCA: myeloperoxidase-anti-neutrophil cytoplasmic antibody; PR3-ANCA: proteinase-3-anti-neutrophil cytoplasmic antibody

**Figure 1:**
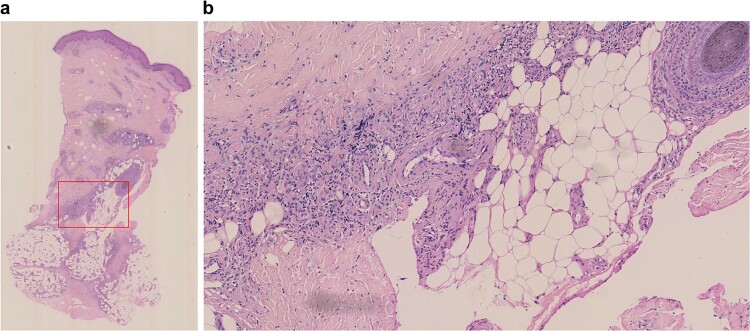
Histopathological findings of subcutaneous nodules on the anterior aspect of the left lower leg before treatment at the public hospital (hematoxylin–eosin staining). Infiltration of numerous lymphocytes and neutrophils around the medium-sized vessels in the deep dermis. a) Low magnification, b) magnification of the red square in A.

**Figure 2:**
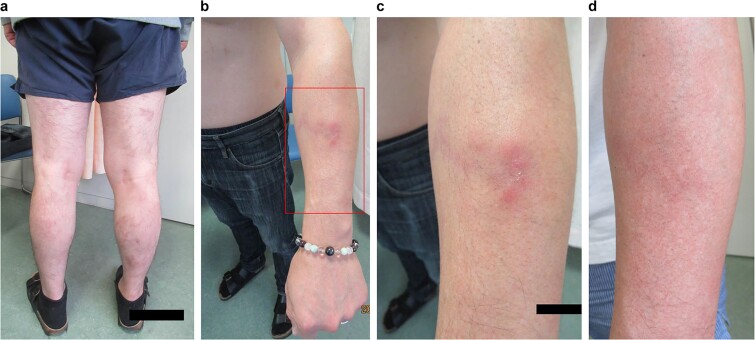
Photographs showing the skin findings on the lower limbs and left forearm. a) At the time of referral to our hospital, posterior aspect of the lower limbs had multiple areas of pigmentation from recurrent rashes. b, c) there was a painful, inflamed, indurated rash on the left forearm (C is an enlarged view of the red square in B). d) the previously noted rash over the left forearm has resolved after treatment with UPA. UPA: Upadacitinib.

**Figure 3:**
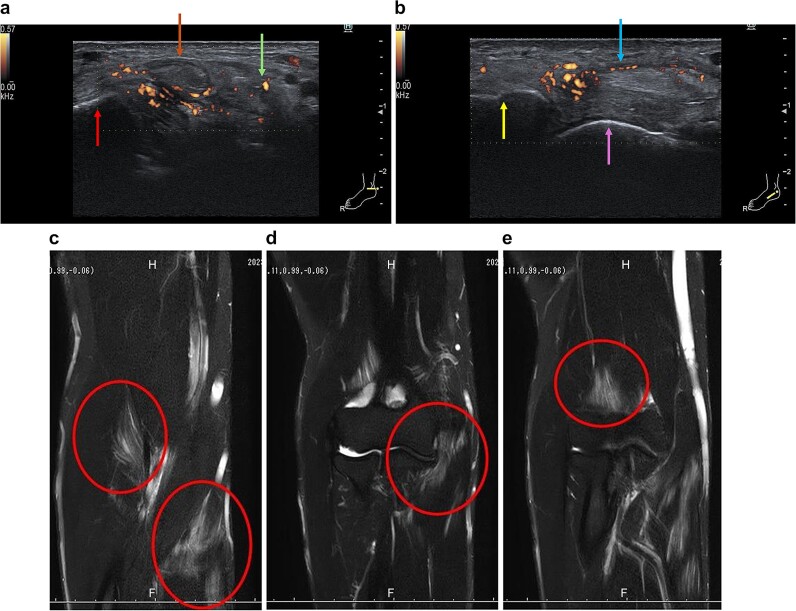
Ultrasound findings of the right ankle joint and magnetic resonance imaging findings of the right elbow at the time of referral to our hospital. a) Signs of tenosynovitis are observed in the posterior tibial tendon and flexor digitorum longus tendon (red arrow: Medial malleolus; brown arrow: Posterior tibial tendon; green arrow: Flexor digitorum longus tendon). b) There were signs of inflammation at the site of insertion of the posterior tibial tendon on the navicular tuberosity (yellow arrow: Navicular tuberosity; purple arrow: Talus; blue arrow: Posterior tibial tendon). c-e) Spectral Attenuated Inversion Recovery (SPAIR) sequence showing high signal intensity in multiple muscles around the right elbow.

**Figure 4:**
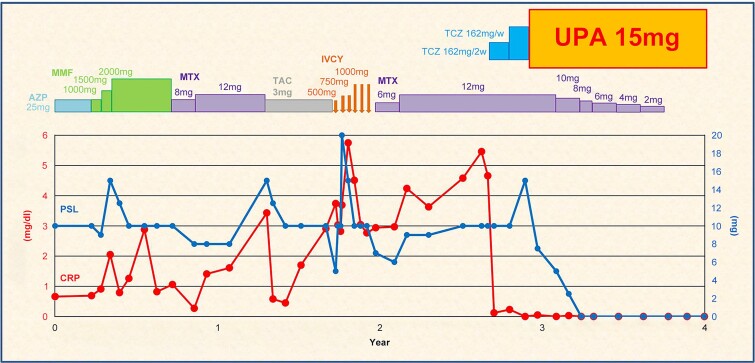
Schematic of the treatment course and the temporal changes in PSL dose and CRP levels since the patient was referred to our hospital. In addition to PSL, immunosuppressants such as AZP, MMF, MTX, TAC, and CY were used; however, intermittent clinical symptoms, including painful subcutaneous nodules, polyarthritis, and myalgia persisted and CRP levels remained elevated. Although administration of tocilizumab normalized CRP levels, there was no improvement in clinical symptoms. Therefore, the dosing interval of tocilizumab was shortened from every 2 weeks to weekly, but clinical symptoms persisted. Thus, UPA was administered, leading to relatively rapid improvement in symptoms, allowing for tapering and discontinuation of MTX, PSL, and NSAIDs (NSAIDs are not depicted in the figure). PSL: Prednisolone; CRP: C-reactive protein; AZP: Azathioprine; MMF: Mycophenolate mofetil; MTX: Methotrexate; TAC: Tacrolimus; CY: Cyclophosphamide; TCZ: Tocilizumab; UPA: Upadacitinib; NSAIDS: Nonsteroidal anti-inflammatory drugs; IVCY: Intravenous cyclophosphamide.

A skin biopsy from a subcutaneous nodule on his left lower leg showed infiltration of numerous lymphocytes and neutrophils in the medium-sized vessels in the deep dermis (Fig. 1). Based on Watts criteria, a diagnosis of PAN was established [3], and treatment with PSL 40 mg was initiated. AZP was included later in the treatment. Five months into treatment, on PSL 10 mg and AZP 25 mg, he was referred to our hospital because of severe pain despite taking NSAIDs three times daily. At the first visit to our hospital, he was 40 years old, 171 cm tall, and weighed 80 kg. His vitals were as follows: blood pressure of 132/74 mmHg; pulse of 91 bpm; respiration rate of 12 breaths/min; SpO_2_ of 97% (room air), and body temperature of 36.0°C. Despite ongoing treatment, he showed no improvement in joint pain, myalgia, and painful rashes (Fig. 2a–c); the CRP level was 0.66 mg/dl. An ultrasound revealed tenosynovitis in the right posterior tibial and flexor digitorum longus tendons, with inflammation at the navicular tuberosity attachment of the posterior tibial tendon (Fig. 3a and b). Moreover, magnetic resonance imaging of the right elbow showed high signal intensity in multiple muscles on spectral attenuated inversion recovery (SPAIR) sequence (Fig. 3c–e). Despite changes in immunosuppressants (Fig. 4), his symptoms and CRP levels did not improve, and PSL could not be tapered.

Although the administration of TCZ every two weeks normalized the CRP levels, he showed no symptomatic improvement. Even when the TCZ dosing interval was reduced to weekly, tapering of PSL was not possible. Subsequently, switching to UPA resulted in a relatively rapid resolution of joint pain, myalgia, and rashes (Fig. 2d). MTX, PSL, and NSAIDs were gradually discontinued, and after one year of UPA use, there were no relapses or side effects.

## Discussion

We report a patient with refractory PAN who presented with rashes, joint pain, and myalgia unresponsive to various immunosuppressants. At present, there is a lack of further treatment options for patients with PAN who do not respond to the various glucocorticoid-immunosuppressant combination therapies recommended by the 2021 American College of Rheumatology/Vasculitis Foundation guidelines [2]. AZP, MMF, MTX, TAC, and CY were ineffective in our patient. Elevated interleukin 6 (IL-6) level in PAN is associated with high disease activity [4]. Previous case reports and case series have indicated that TCZ may be effective for refractory PAN [5]. Therefore, TCZ was used but was ineffective in this case.

There is only one report of tofacitinib (TOF) being effective for refractory systemic PAN [6], and one case series report has described its effectiveness in four cases of refractory cutaneous PAN [7]. Another case report has documented the effectiveness of TOF in a refractory case of leukocytoclastic vasculitis, which is another type of vasculitis [8].

Considering that UPA has the most indications among JAK inhibitors and is most frequently used at our facility, we chose UPA after TCZ. This led to relatively rapid improvement in clinical symptoms, allowing for the tapering and discontinuation of MTX, PSL, and NSAIDs (Fig. 4). After one year, no relapses or side effects were noted.

The reason for the effectiveness of UPA is unclear. Rimar et al. reported a patient with refractory PAN in whom TCZ was ineffective, but TOF was effective. They suggested that in PAN, the JAK-STAT3 (Signal transducer and activator of transcription 3) pathway might be directly stimulated by factors bypassing the IL-6 pathway, such as Toll-like receptor 4, 7, 9, and interleukin 23 [6]. Similar to TOF, UPA blocks multiple cytokine pathways, possibly explaining its effectiveness in this case.

Moreover, there is emerging evidence that JAK inhibitors are effective in patients with difficult-to-treat rheumatoid arthritis (D2TRA) [9], suggesting potential effectiveness for refractory PAN similar to D2TRA. Owing to the rarity of PAN, conducting randomized controlled trials is challenging. Accumulating case reports on JAK inhibitors is crucial for future research.
